# Predictive factors and early biomarkers of response in multiple sclerosis patients treated with natalizumab

**DOI:** 10.1038/s41598-020-71283-5

**Published:** 2020-08-28

**Authors:** Maria Inmaculada Dominguez-Mozo, Silvia Perez-Perez, Luisa María Villar, Begoña Oliver-Martos, Noelia Villarrubia, Fuencisla Matesanz, Lucienne Costa-Frossard, María Jesús Pinto-Medel, María Isabel García-Sánchez, Isabel Ortega-Madueño, Lorena Lopez-Lozano, Angel Garcia-Martinez, Guillermo Izquierdo, Óscar Fernández, Jose Carlos Álvarez-Cermeño, Rafael Arroyo, Roberto Alvarez-Lafuente

**Affiliations:** 1 Grupo de Investigación de Factores Ambientales en Enfermedades Degenerativas, Pabellón B. Laboratorio Investigación Esclerosis Múltiple, Instituto de Investigación Sanitaria del Hospital Clínico San Carlos (IdISSC)/Hospital Clínico San Carlos, Red Española de Esclerosis Múltiple (REEM), C/ Martín Lagos S/N, 28040 Madrid, Spain; 2 Servicio de Inmunología, Hospital Universitario Ramón Y Cajal/Instituto Ramón y Cajal de Investigación Sanitaria (IRYCIS), Red Española de Esclerosis Múltiple (REEM), Madrid, Spain; 3 UGC Neurociencias. Hospital Regional Universitario de Málaga/Instituto de Biomedicina de Málaga (IBIMA), Red Española de Esclerosis Múltiple (REEM), Madrid, Spain; 4 Departmento de Biología Celular E Inmunología, Instituto de Parasitología Y Biomedicina López Neyra (IPBLN)/Consejo Superior de Investigaciones Científicas (CSIC), Red Española de Esclerosis Múltiple (REEM), Madrid, Spain; 5 UGC Neurología (Biobanco Hospitalario), Hospital Universitario Virgen Macarena, Red Española de Esclerosis Múltiple (REEM), Madrid, Spain; 6 Servicio de Análisis Clínicos, Hospital Clínico San Carlos, Madrid, Spain; 7 UGC Neurología, Hospital Universitario Virgen Macarena, Red Española de Esclerosis Múltiple (REEM), Madrid, Spain; 8 Departamento de Neurología, Hospital Universitario Quironsalud Madrid, Red Española de Esclerosis Múltiple (REEM), Madrid, Spain

**Keywords:** Immunology, Microbiology, Molecular biology, Neuroscience, Biomarkers, Neurology

## Abstract

There are an increasing number of treatments available for multiple sclerosis (MS). The early identification of optimal responders to individual treatments is important to achieve individualized therapy. With this aim, we performed a multicenter retrospective longitudinal study including 186 MS patients treated with natalizumab who were followed for 2 years. We analyzed the following variables at recruitment: sex, current age, age at disease onset, disease duration, EDSS, number of T2 and Gd + lesions, IgG and IgM oligoclonal bands, HLA class II (DR, DRB, DQA, DQB, and DRB1*15:01), IgG and IgM antibody titers against human herpesvirus 6 (HHV-6) and the antibody response to Epstein–Barr virus (EBV) through the measurement of the anti-EBNA-1 and anti-VCA IgG titers, in relation to clinical response (no relapses or disability progression), and to NEDA-3 (no evidence of disease activity in terms of clinical response and no changes in MRI scans either) after 2-years follow-up. Baseline EDSS score, baseline EBNA-1 IgG titers and percentage change of HHV6 IgG titers between baseline and 6 month visits were significantly different in clinical responders and in NEDA-3 status (all of them remained significant in the multivariate analysis). We identified three variables for the early identification of natalizumab optimal responders in a rapid and cost-effective approach.

## Introduction

In the last years, there are an increasing number of treatments available for multiple sclerosis (MS) patients. Since natalizumab, a humanized monoclonal antibody against the cell adhesion molecule α4-integrin^1^, was approved by the U.S. Food and Drug Administration (FDA) in 2004 to treat MS, several treatments or new formulations have also been approved to treat this disease: fingolimod, teriflunomide, alemtuzumab, dimethyl fumarate, pegilated interferon beta-1a, ocrelizumab and cladribine^2^. Therefore, to identify in an early stage the most appropriated treatment is of great importance to avoid treatment failures that could negatively affect the evolution of the disease, to choose cost-effective treatments to optimize financial resources and to avoid possible secondary effects that could be life threatening.

With this aim, we searched in a retrospective study for possible predictive factors and biomarkers of response in MS patients treated with natalizumab.

## Results

### Patients eligible for the study and demographic characteristics of the population study

A total of 381 MS patients have been treated with natalizumab or were under natalizumab treatment (with at least one intravenous infusion and with at least one serum sample collected) when we started this retrospective study: 109 MS patients were currently under natalizumab treatment for less than two years, 6 abandon natalizumab treatment for pregnancy (planning or confirmed), in 14 MS patients treatment was withdrawn due to disease activity before reaching 2 years, in 25 MS patients treatment was withdrawn for other reasons (lack of tolerance, adverse event, risk of LMP due to JCV + serology, patient choice,…), and for 41 MS patients we did not have any of the required serum samples or clinical/radiological data. Finally, 186 MS patients fulfilled all the inclusion criteria; their characteristics are shown in Table 1.

**Table 1:** Demographical characteristics of the patients included in the study at the onset of natalizumab treatment.

**Gender**	**N**
Males	64
Females	122
**Age (years, med (P25-P75))**	37.0 (27.0–46.5)
**Age at disease onset (years, med (P25-P75))**	27.0 (19.0–38.0)
**Disease duration at natalizumab onset (months, med (P25-P75))**	92.0 (24.0–226.0)
**MS type**	**N**
RR	169
SP	17
**EDSS (med (P25-P75))**	3.0 (1.5–5.5)
**Relapses 2 years before (med (P25-P75))**	2.0 (1.0–4.5)
**Treatment naïve (N)**	28
**Last treatment before natalizumab onset**	**N**
Glatiramer acetate	54
Interferon beta	93
Mitoxantrone	7
Cyclophosphamide	2
Fingolimod	1
Teriflunomide	1
**Duration of the last treatment (months, med (P25-P75))**	25.0 (11.0–49.0)
**Number of previous treatments before natalizumab onset**	**N**
1	86
2	49
3	15
4	6
5	2
Months treated before natalizumab onset (med (P25-P75))*	49.0 (21.0–81.0)
**Patients receiving previous immunosuppressant treatment (N)**	19
Months under immunosuppressant treatment (med (P25-P75))**	15.0 (3.0–24.0)
**Months since disease onset until first treatment (med (P25-P75))**	32.0 (13.0–83.0)

*med* median, *P25* 25th percentile, *P75* 75th percentile, *EDSS* Expanded Disability Status Scale.

*Only among those MS patients who received at least one treatment before natalizumab onset.

**Only among those MS patients who received this treatment.

### Clinical and radiological response after two years of natalizumab treatment

The relapse rate was 0.4 (27.5% of MS patients suffered relapses) vs. 2.4 two years prior to natalizumab onset (83.3% of reduction). The mean variation in the EDSS was − 0.1 (12.9% of MS patients experienced progression; in 34.4% EDSS decreased). Regarding the MRI studies, the 18.3% (34/186) of patients had new T2 lesions after 12-months of natalizumab treatment and only the 3.8% (7/186) in the second year; the 7.0% (13/186) had Gd + lesions at 12-month MRI and only the 2.7% (5/186) of patients had Gd + lesions at 24-month MRI. According to our response criteria, the 63.4% could be considered as clinical responders and the 43.5% as NEDA-3 after 2 years of natalizumab treatment.

### Clinical and radiological variables as early markers of response to natalizumab treatment

We only found an association for the baseline EDSS. We found that 77.9% of patients with baseline EDSS < 3 (median value) could be considered as clinical responders vs. 48.1% of those with baseline EDSS > 3 (p = 0.002; O.R. = 3.8). Likewise, 67.9% of MS patients with baseline EDSS < 3 showed NEDA-3 after two years of natalizumab treatment vs. 35.8% of those with baseline EDSS > 3 (p = 0.006; O.R. = 3.8). Finally, when we analyzed the therapeutic failure, 32.9% (26/79) of patients with baseline EDSS > 3 experienced progression and/or more than one relapse vs. 11.6% (10/86) of patients with baseline EDSS < 3 (p = 0,001; O.R. = 3.7).

### Oligoclonal bands (OCBs) as early biomarkers of response to natalizumab treatment

A total of 158/186 MS patients had data about their IgG-OCBs and 91/186 about IgM-OCBs. The 88.6% (140/158) was positive for IgG-OCBs and the 60.4% (55/91) for IgM-OCBs. We did not find any statistical association between the presence or absence of IgG or IgM OCBs and the clinical response to natalizumab (89/140 IgG-OCBs + vs. 9/18 IgG-OCBs-, p = 0.264, and 36/55 IgM-OCBs + vs. 21/36 IgM-OCBs-, p = 0.492, were responders) or the NEDA-3 status (61/140 IgG-OCBs + vs. 7/18 IgG-OCBs-, p = 0.706, and 25/55 IgM-OCBs + vs. 13/36 IgM-OCBs-, p = 0.377, reached NEDA-3 condition after 2 years of follow-up).

### HLA-II as early marker of response to natalizumab treatment

After Bonferroni correction, we only found some trends for the HLA-DQB1-201 in relation with new T2 lesions, Gd + lesions and NEDA-3 condition and for HLA-DQB1-202 also with NEDA-3 status (p = 0.0118, p = 0.0022, p = 0.0050 and p = 0.0377 before Bonferroni correction, respectively).

### Baseline viral serologies as early biomarkers of response to natalizumab treatment

We found statistical significant differences for EBNA-1 IgG, but not for VCA IgG or HHV-6 IgG and IgM. A p value of 0.042 was found with the Kruskal–Wallis test for the clinical response (p = 0.053 for the NEDA-3 condition). Further analysis (two-tailed Fisher’s exact test) showed a p value of 0.018 when we analyzed the clinical response in patients with EBNA-1 titers above and below the median value (23.3 AU) (p = 0.032 for the NEDA-3 condition). Furthermore, patients with the lowest titers (4th quartile; < 21.5 AU) were more prone to be clinical responders (35/43; 81.4%) than those with the highest titers (1st quartile; > 25.5 AU) (21/43; 48.8%): p = 0.002; O.R. = 4.6 (p = 0.01 for the NEDA-3 condition).

### Combination of variables showing significant associations at baseline visit

We explored the combination of the two variables showing significant associations in the univariate analysis (baseline EDSS and EBV baseline titers above and below median values) as predictive factors of clinical response. Results are shown in Table 2.

**Table 2:** Comparison between MS patients with both variables showing significant associations at baseline visit vs. MS patients without both of them.

Baseline EDSS	Baseline EBNA-1 IgG titers	NEDA-3	Clinical responders	Therapeutic failure
< 3.0 (1.0–2.5)	< 23,3 AU	28/43 (65.1%)	34/43 (79.1%)	2/43 (4.6%)
> 3.0 (3.5–7.5)	> 23,3 AU	8/35 (22.9%)	14/35 (40.0%)	13/35 (37.1%)
	p value	0.0002	0.0004	0.0003
	O.R	6.3	5.7	12.1

### Variation of the viral serologies in the first 6 months of natalizumab treatment

**Figure 1:**
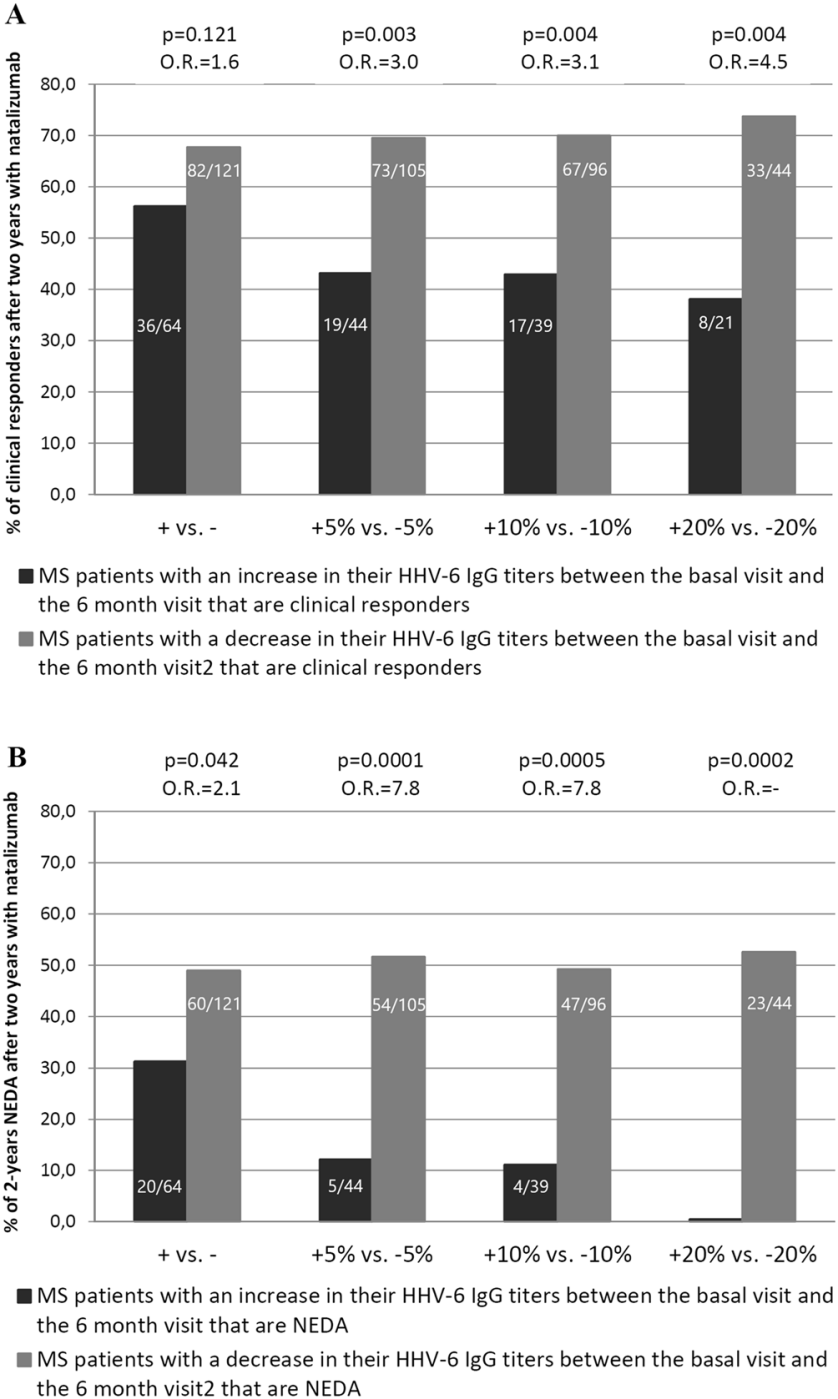
Percentage of clinical responders (**A**) and 2-years NEDA-3 (**B**) MS patients when comparisons are made between the same percentage of increase or decrease of HHV-6 IgG titers between the baseline visit and the six month visit.

The decrease of the HHV-6 IgG antibody titers after six months of treatment was statistically associated with the response to natalizumab: p = 0.010 for the clinical response and p = 0.003 for 2-years NEDA-3 condition (Kruskal–Wallis test). The median values of the HHV-6 titers were 25.0 AU for the baseline samples and 22.1 AU for the six month samples (25.9 AU and 21.0 AU, for clinical responders vs. 24.4 AU and 23.1 AU for non-responders, respectively; 25.9 AU and 21.2 AU, for patients that reached NEDA-3 condition vs. 24.4 AU and 22.8 AU for patients that did not reach this condition after 2 years of follow-up, respectively). It was the only significant association. Furthermore, when we compared the percentage of clinical responders and 2-years NEDA-3 patients as we performed in our previous publication^3^, we found similar results with only six-months of follow-up (Fig. 1). As we can see in Fig. 1B, we did not find NEDA-3 patients when increases in the HHV-6 IgG titers were higher than 20%.

### Combination of variables showing significant associations at baseline visit and after 6 months of treatment

We performed a combination of the two variables showing significant associations in the univariate analysis at baseline visit (baseline EDSS and EBV baseline titers above and below median values) and at 6 month visit (the decrease of the HHV-6 IgG antibody titers). Results are shown in Table 3.

**Table 3:** Combination of the three variables with statistical significant associations at baseline visit or at six month visit.

	Baseline EDSS*	Baseline EBNA-1 IgG titers**	HHV-6 variation (0–6 months)***	NEDA-3	Clinical responders	Therapeutic failure
(All variables below the first quartile)	< 2.0 (1.0–1.5)	< 21.5 AU	> 20% of decrease	75.0%	100.0%	0.0%
(All variables below the median value)	< 3.0 (1.0–2.5)	< 23.2 AU	> 5% of decrease	65.2%	85.2%	3.7%
(All variables above the median value)	> 3.0 (3.5–7.5)	> 23.2 AU	< 5% of decrease	23.5%	40.9%	31.8%
(All variables above the fourth quartile)	> 4.0 (4.5–7.5)	> 25.5 AU	> 5% of increase	0.0%	0.0%	60.0%

*Baseline EDSS: 1st quartile: 2.0; median value: 3.0; 4th quartile: 4.0.

**Baseline EBNA-1 IgG titers: 1st quartile: 21.5 AU; median value: 23.2 AU; 4th quartile: 25.5 AU.

***HHV-6 variation between IgG titers of the baseline visit and the six months visit: 1st quartile: 20% of decrease; median value: 5% of decrease; 4th quartile: 5% of increase.

### Multivariate analysis

When we considered the clinical response, only baseline EDSS (p = 0.002; O.R. = 0.738 (IC95%: 0.608, 0.896)) and the percentage of variation of the HHV6 IgG titers (p = 0.035; O.R. = 0.986 (IC95%: 0.974, 0.999)) remained significant in the multivariate analysis. However, when we analyzed patients who reached or not NEDA-3 status, the three variables with significant associations in the univariate analysis remained significant: baseline EDSS (p = 0.022; O.R. = 0.747 (IC95%:0.582, 0.959)), baseline EBNA-1 IgG titers (p = 0.038; O.R. = 0.920 (IC95%:0.851, 0.995)), and percentage of variation of the HHV6 IgG titers (p = 0.002; O.R. = 0.968 (IC95%: 0.949, 0.988)).

### Sub-analysis in secondary progressive MS cohort

Finally, despite the small sample size (only 17 patients), we performed a sub-analysis of the same epidemiological, clinical, radiological, serological and genetic variables in the secondary progressive (SP) MS cohort. We found a trend between the HHV-6 IgG antibody variation and the NEDA-3 status: the mean variation among MS patients with NEDA-3 was − 38.5% vs. 0.2% among SPMS patients without NEDA-3 (p = 0.060). Similarly, only for those SPMS with the highest baseline HHV-6 IgG and IgM titers we found a trend with the clinical response (p = 0.083 and p = 0.036, respectively). No other trends or significant differences were founds for any one of the variables analyzed.

### Variables of response in the cohort of MS patients in which treatment was withdrawn due to disease activity before reaching two years

The treatment was withdrawn in 5/14 at 6 months due to relapses and progression (only one serum sample was collected at this point). In 7/14 treatment was withdrawn between 10–15 months due to progression (3/14), progression plus relapse (1/14) and new T2 lesions at MRI (3/14). In 2/14 natalizumab was withdrawn after 18 and 19 months of treatment due to progression and a severe relapse, respectively. When we analyzed the three variables of response that we have described: 8/14 had a baseline EDSS > 3.0 and 2/14 had an EDSS = 3.0; in 5/13 the baseline titers of EBNA-1 was in the 1st quartile (> 25.5 AU) vs. only 1/13 in the 4th quartile (< 21.5 AU); and 7/10 experienced an increase of the HHV-6 IgG antibody titers after six months of treatment vs. 1/10 that had the same titers, 1/10 with a reduction < 5% and 1/10 with a reduction > 5%.

## Discussion

The development of predictive models of response to the different treatments is something crucial in the modern medicine. The phrase “time is brain” that was coined for the acute thrombolytic treatment of ischemic stroke is also relevant in MS. This phrase not only comprises the early initiation of a first line therapy but also the monitoring of disease activity under therapy to switch as early as possible to another treatment if a suboptimal response is detected^4^. Therefore, the identification of predictive factors and early biomarkers is of great importance to avoid future treatment failures.

The detection of neutralizing antibodies against natalizumab have been associated with a reduction in treatment efficacy^5^. Anti-JC virus (JCV) antibody index^6^, and lipid-specific immunoglobulin M bands in cerebrospinal fluid^7^ could allow risk stratification for the development of progressive multifocal leukoencephalopathy (PML), a rare but severe adverse event during natalizumab treatment. Regarding clinical predictors, in a study of Prosperini et al.^8^ with 210 MS patients that completed 24-month of follow-up the authors found two predictive variables for having a full response to natalizumab: less than 2 relapses in the previous year and an EDSS score < 3.0 at baseline. Another study published one year later by Sargento-Freitas et al.^9^, with a small cohort of 48 MS patients treated with natalizumab, confirmed that lower baseline EDSS score associates with optimal response to natalizumab; in contrast, they showed that a higher relapse rate in the year prior to natalizumab initiation associated with an optimal response to natalizumab. Our data confirm that a baseline EDSS score below 3.0 is a predictive factor for a good clinical response and also for reaching the NEDA-3 status. The confirmation of this biomarker in three independent cohorts, demonstrate that it would be very useful in clinical practice to establish accurate treatment decisions. This was not the case with the previous annualized relapse rate. Previous studies offered contradictory results and we did not find any significant differences in our study either.

Apart from the baseline EDSS, we also describe here two putative biomarkers of response to natalizumab: anti EBNA-1 IgG antibody titers in baseline samples and the variation in the HHV-6 IgG titers between baseline and the six month samples. Furthermore, the three biomarkers are easy to obtain, accessible and affordable to almost all the hospitals, rapid and cost effective.

The correlation between the variation of the HHV-6 IgG antibody titers between baseline and 6 month visits and the response to natalizumab in this multicenter study corroborates our previous report, when changes between baseline and two years visits were analyzed^3^. Our current data support the role of these antibodies in predicting patient response and provide an earlier biomarker by assessing these changes at six month visit.

The correlation of the baseline titers of EBNA-1 IgG antibodies and the variation of the HHV-6 IgG antibodies with the response to natalizumab could help us to better understand the possible role of these viruses in the pathogenesis of the disease. A possible explanation for these results could be found in the literature: higher titers of HHV-6 IgG antibodies have been previously related with relapses by our group^3^ and others^10^. This may only be a reflection of the exacerbation of the inflammatory status of MS patients before the relapses, or it could be related to the possible participation of this virus in the pathogenesis of the disease by one of the mechanisms that have been previously proposed, or both at the same time. Anyway, since higher titers of HHV-6 IgG antibodies seem to be related to the occurrence of relapses in the disease and treatment with natalizumab drastically reduces the relapse rate in MS patients, it would be expected that those responding patients show a reduction in the titers of the antibodies against this virus. This is what we describe here only for HHV-6 IgG antibodies variation, but not for the variation in the antibody response to EBV (EBNA-1 or VCA).

Regarding EBV, the association of this virus and relapses in MS is unclear^11,12^. However, EBV has been postulated as a primary cause of neurodegeneration in MS due to the presence of EBV infected B cells in CNS and the external immune reaction driven by EBV replication or by EBV-infected B cells presenting CNS antigens^13^. Higher neurodegeneration implies higher EDSS scores, a predictive factor of bad response to natalizumab, as it has been described here and by others^8,9^. Thus, this would explain the results found in the present study between having low baseline titres of anti-EBV antibodies and a better clinical response to natalizumab. This also could explain why we did not find any correlation for baseline titres of anti-EBV in the SPMS cohort (all of them with EDSS > 4.0).

One of the possible limitations of the study is the absence of a re-baseline MRI; thus, the significance of the appearance of a single new T2 lesion in the 12-month MRI compared with that performed one month before natalizumab initiation could be unclear. An inclusion bias was also introduced excluding those MS patients with ongoing disease activity that switched treatment before the 2 year time point; although the results found in this cohort for the three variables described are consistent with those found in the study cohort, future studies should take this into account. Finally, since the paper by Engdahl et al. analyzing the serology of HHV-6A and HHV-6B in MS patients was published^14^, it would be very interesting to know if the observed association between the response to natalizumab and the variation of the HHV-6 titers in the first 6 months is due to one of these two viruses or not, which could also have important consequences regarding their possible involvement in the etiopathogenesis of the disease.

In conclusion, our results describe three variables capable of identifying MS patients with high probability of being optimal responders to natalizumab, which can be useful in daily clinical practice.

## Materials and methods

### Design

This is a retrospective study. Inclusion criteria: MS patients diagnosed by Poser^15^ or 2010 McDonald^16^ criteria with: (1) natalizumab treatment for at least 2 years; (2) a serum sample collected within a month before treatment onset and a second serum sample 6 months after initiation; (3) clinical (EDSS and relapse rate) and radiological (T2 lesions and Gd + lesions) data at initiation, and 1 and 2 years later; (4) an analysis of the antibodies against natalizumab. Exclusion criteria: MS patients that had transient or persistent antibodies against natalizumab.

### Patients

Patients belonged to the following hospitals: Hospital Clínico San Carlos (Madrid), Hospital Universitario Ramón y Cajal (Madrid), Hospital Regional Universitario (Málaga) and Hospital Universitario Virgen Macarena (Sevilla), all of them from Spain. All clinical data were collected by neurologists of the Multiple Sclerosis Units of those hospitals.

### Response criteria

Progression was defined depending on pre-treatment EDSS score: (1) increase ≥ 1.5 points at 24-months visit if pre-treatment EDSS = 0; (2) increase ≥ 1 point at 24-months visit if pre-treatment EDSS was ≥ 1 and ≤ 5; (3) increase ≥ 0.5 points at 24-months visit if pre-treatment EDSS was ≥ 5.5. Relapses were considered as a worsening of neurological impairment or an appearance of a new symptom or abnormality attributable to MS, lasting at least 24 h and preceded by stability of at least 1 month. Magnetic resonance imaging (MRI) of the brain was performed one month prior natalizumab onset and 1 and 2 years after starting this therapy in 1.5 T scanners following a previous published protocol^17^. The sequences collected for this study were axial T2-weighted imaging, axial fluid-attenuated inversion recovery (FLAIR) T2, axial proton density T2-weighted imaging, and T1-weighted imaging with Gd enhancement. Slice thickness of 5 mm was acquired to obtain contiguous axial sections that covered the entire brain. In each center, the same machine was used for the three follow-up visits of the patients included in the study (prior natalizumab onset and 1 and 2 years after starting this therapy), and the exams were reported by the same radiologists.

With the previous definitions, we establish the following response criteria after two years of follow-up: clinical response (defined as an absence of relapses and disability progression), NEDA-3 (no evidence of disease activity: no relapses, no disability progression, with no new T2 lesions or Gd + lesions) and therapeutic failure (≥ 2 relapses and/or disability progression).

### Researched variables

We analyzed the following variables:

- Clinical: gender, age at disease onset, age at recruitment, disease duration, previous treatments, EDSS score at treatment initiation.
- Radiological: number of T2 and Gd + lesions in the MRI performed at recruitment.
- Oligoclonal bands: IgG and IgM.
- Genetic: HLA I and II.
- Environmental: antibody responses to EBV (EBNA-1 and VCA) and anti HHV-6 IgG and IgM antibody titers in baseline serum samples, as well as their changes between the baseline and six month sample, based on previous results published by our group^3^.

### DNA extraction

Total DNA was isolated by DNA spin column technique of QIAamp DNA Blood Mini Kit (QIAGEN. Hilden. Germany), from 0.2 ml of blood, and QIAamp Ultrasens Virus Kit (QIAGEN), from 1 ml of serum, according to the manufacturer’s instructions. Each sample was extracted in duplicate. Two negative controls (water free of nucleases and proteases), were included with each set of eight samples, as we have previously published^18^.

### Neutralizing antibodies against natalizumab

The detection and confirmation of natalizumab antibodies in human serum samples collected at the third month of treatment was made by ELISA as described previously^19^.

### Oligoclonal bands detection

The detection of the IgG and IgM OCBs was performed by isoelectric focusing and Western blot as it has been previously described^20^.

### HLA genotyping

HLA class II (DR, DRB, DQA, DQB) was genotyped using SSOP technology (Sequence specific-oligonucleotid probe); HLA DRB1*15:01was analyzed by Taqman technology in a 7900HT Fast Real-Time PCR system, under the conditions recommended by the manufacturer (Applied Biosystems, Foster City, CA, USA).

### ELISA

Every serum sample was tested with commercial tests for the detection of anti-HHV-6A/B IgG and IgM (Vidia, Ltd., Czech Republic), and anti-EBNA-1 and anti-VCA IgG (Trinity Biotech, USA), following manufacturer instructions, in an automated ELISA processing system (DS2, Dynex Technologies, USA). As we have previously published^3,16^, results were expressed in artificial units (AU); they were calculated multiplying the index value by 10 (index value = sample absorbance/cut-off value). Samples were analyzed in duplicate for each test, and doubtful samples, those that were between 9 and 11 AU were tested again; if the re-tested samples were below 11 AU they were considered as negative.

### Statistical analysis

The chi-square or two-tailed Fisher’s exact test was used to test differences in categorical variables. Kruskall–Wallis analysis or the Wilcoxon rank-sum test was used to test differences in continuous variables. We used a multiple logistic regression model to eliminate the effect of the putative confounding variables in the univariate tests. We also studied the interaction between baseline EDSS and EBNA-1 baseline titles. P-values < 0.05 were considered as statistically significant. All analyses were performed using SPSS for Windows (Ver. 15.0) software (SPSS Inc.).

### Ethics statement

This study was approved by local Ethic Committees of the four centers: Comité Ético de Investigación Clínica del Hospital Clínico San Carlos, Comité Ético de Investigación Clínica del Hospital Universitario Ramón y Cajal, Comité de Ética de la Investigación del Hospital Regional Universitario Carlos Haya de Málaga y Comité de Ética de la Investigación de Centro de los Hospitales Universitarios Virgen Macarena—Virgen del Rocío de Sevilla. All the patients recruited received and signed a written informed consent. All experiments were performed in accordance with relevant guidelines and regulations.
